# Recursive Exponentially Weighted N-way Partial Least Squares Regression with Recursive-Validation of Hyper-Parameters in Brain-Computer Interface Applications

**DOI:** 10.1038/s41598-017-16579-9

**Published:** 2017-11-24

**Authors:** Andrey Eliseyev, Vincent Auboiroux, Thomas Costecalde, Lilia Langar, Guillaume Charvet, Corinne Mestais, Tetiana Aksenova, Alim-Louis Benabid

**Affiliations:** 1 Univ. Grenoble Alpes, CEA, LETI, CLINATEC, MINATEC Campus, 38000 Grenoble, France; 2 Centre Hospitalier Universitaire Grenoble Alpes, 38700 La Tronche, France

## Abstract

A tensor-input/tensor-output Recursive Exponentially Weighted N-Way Partial Least Squares (REW-NPLS) regression algorithm is proposed for high dimension multi-way (tensor) data treatment and adaptive modeling of complex processes in real-time. The method unites fast and efficient calculation schemes of the Recursive Exponentially Weighted PLS with the robustness of tensor-based approaches. Moreover, contrary to other multi-way recursive algorithms, no loss of information occurs in the REW-NPLS. In addition, the Recursive-Validation method for recursive estimation of the hyper-parameters is proposed instead of conventional cross-validation procedure. The approach was then compared to state-of-the-art methods. The efficiency of the methods was tested in electrocorticography (ECoG) and magnetoencephalography (MEG) datasets. The algorithms are implemented in software suitable for real-time operation. Although the Brain-Computer Interface applications are used to demonstrate the methods, the proposed approaches could be efficiently used in a wide range of tasks beyond neuroscience uniting complex multi-modal data structures, adaptive modeling, and real-time computational requirements.

## Introduction

The Brain-Computer Interface (BCI) measures and processes the brain’s neural activity to provide a patient with a non-muscular communication pathway to external devices such as robotic arms, wheelchairs, or exoskeletons^1–4^. BCI systems could be used to rehabilitate and improve the quality of life of individuals with severe motor disabilities^5,6^. To register the neural activity, electroencephalography (EEG)^1,7^, electrocorticography (ECoG)^8–11^, microelectrodes array^12,13^, and magnetoencephalography (MEG)^14–16^, etc. are applied in BCIs.

The high dimensionality of the data is unique to neural activity processing^17^. Partial Least Squares (PLS) regression^18,19^ and Principal Component Analysis (PCA)^20^ are particularly appropriate for high dimension tasks. In contrast to PCA, PLS considers both the exploratory and response variables by projecting them to the low dimensional space of latent variables. Statistical properties are considered for both vector^21^ and multi-way (tensor)^22^ versions of PLS. Due to its efficient dimensional reduction technique PLS-family methods are often used for BCIs^11,23–25^.

While most state-of-the-art approaches for a BCI decoder identification are vector oriented^11,25–31^, it was shown^32^ that multi-way (tensor) data processing could significantly improve the quality of analysis because it considers the intrinsic nature of the data under analysis. The tensor-based approaches allow simultaneous treatment of several domains (modalities), e.g., temporal, frequency, and spatial, for BCI applications. Moreover, the influence of each modality on the prediction model could be studied^33^. In addition, different modalities could be independently penalized to introduce special properties to the modeling tasks, e.g., a sparse solution in the spatial domain for selection of the most informative electrodes subset^34^. Finally, as demonstrated previously^35^, the tensor-based methods could improve the noise-robustness versus the vector-oriented approaches that is of great importance for BCI tasks. To date, multi-way methods were efficiently applied in several BCI systems^36–39^.

The variability of the neuronal signals requires regular recalibration of the BCI systems^35,40,41^. While full system recalibration is computational and time consuming, adaptive adjustment of the previously calibrated model could successfully process mild changes in the brain signals. Different adaptive methods were proposed and shown to be efficient for both EEG-based^41–45^, ECoG-^35,46^ and microelectrode array-based^12^ BCI systems.

Adaptation is efficient in the case of long periods of signal observation because identifies the model without storing it in the memory over entire training sets. Moreover, continuous adjustment of the predictive model allows reaction to changes in the subject’s behavior with minimal delay. This is critically important for the training process^47,48^.

Several vector-oriented PLS-family recursive algorithms, e.g. Recursive PLS (RPLS)^49,50^ and Recursive Exponentially Weighted PLS (REW-PLS)^51,52^ were proposed for adaptive modeling. Adaptive PARAFAC tensor-based algorithms were suggested recently^53,54^. Recursive Multi-way PLS (RNPLS) was proposed^35^ as a generalization of the RPLS algorithm to the case of tensor-input/tensor-output variables. However, a common shortcoming of both RPLS and RNPLS methods consists in only part of informative features being stored in each subsequent iteration. This could lead to a loss of quality in the predictive model. On the other side, although the REW-PLS algorithm does not have this shortcoming, it is vector-oriented and cannot efficiently treat the multi-modal data.

Here, we propose a Recursive Exponentially Weighted Multi-way PLS (REW-NPLS) algorithm that is a generalization of the REW-PLS method to the tensor case. Similar to the REW-PLS algorithm, the REW-NPLS does not suffer from a loss of data. Moreover, the REW-NPLS inherits computational efficiency of the REW-PLS, which, as it was demonstrated in^52^, has superior computational performance in comparison to RPLS-based methods. This is especially important for real-time applications. Concurrently, the REW-NPLS approach has all the advantages of tensor-based methods.

All PLS-family methods are iterative and require estimation of the iterations number (factors number) hyper-parameter. Although there is no theoretically-justified algorithm for choice of the optimal value, heuristic approaches are effective in the practical applications, e.g., in the cross-validation procedure^50,55,56^. However, cross-validation is proposed for offline-oriented tasks. It is computationally time-consuming and requires simultaneous access to the entire training set, which is not always possible for online tasks due to the memory limitations. Moreover, offline estimation of hyper-parameters does not allow their adjustment in time. Thus, the possibility to do online estimation and adaptation of the hyper-parameters are important for online tasks. Here, we proposed the Recursive-Validation procedure for hyper-parameters estimation and adjustment, which is particularly well-suited for data flow tasks. The method was integrated to the REW-NPLS algorithm and was compared to the standard “offline” cross-validation approach. The comparison demonstrates equivalent prediction accuracies for both online and offline methods.

The methods proposed here were studied with applications in brain signal decoding, however, they also could be used in a wide range of tasks beyond neuroscience where online data flows of complex structure are processed: e.g., images and video sequences analysis and adaptive monitoring of complex industrial processes.

## Methods

### Generic PLS and NPLS regressions

Ordinary Partial Least Squares regression^18^ is an iterative procedure to estimate a linear relationship between a vector of independent (input) variables and a vector of dependent (output) variables on the basis of observation matrices ${\bf{X}}\in {{\mathbb{R}}}^{N\times n}$ and ${\bf{Y}}\in {{\mathbb{R}}}^{N\times m}$: ${\bf{Y}}={\bf{X}}{\bf{B}}+{\bf{D}}$, where ${\bf{B}}\in {{\mathbb{R}}}^{n\times m}$ is the matrix of linear coefficients, and ${\bf{D}}\in {{\mathbb{R}}}^{N\times m}$ is the matrix of noise. To build the model, the observations are iteratively projected into the low dimensional spaces of latent variables while trying to explain the maximum of variance of ${\bf{X}}$ and ${\bf{Y}}$ simultaneously: ${\bf{X}}={\bf{T}}{{\bf{P}}}^{T}+{\bf{E}}$, ${\bf{Y}}={\bf{U}}{{\bf{Q}}}^{T}+{\bf{F}}$; where ${\bf{T}}=[{{\bf{t}}}_{1},\ldots ,{{\bf{t}}}_{F}]\in {{\mathbb{R}}}^{N\times F}$ and ${\bf{U}}=[{{\bf{u}}}_{1},\ldots ,{{\bf{u}}}_{F}]\in {{\mathbb{R}}}^{N\times F}$ are the matrices of the latent variables (scores), ${\bf{P}}=[{{\bf{p}}}_{1},\ldots ,{{\bf{p}}}_{F}]\in {{\mathbb{R}}}^{n\times F}$ and ${\bf{Q}}=[{{\bf{q}}}_{1},\ldots ,{{\bf{q}}}_{F}]\in {{\mathbb{R}}}^{m\times F}$ are the matrices of the loading vectors (projectors), ${\bf{E}}$ and ${\bf{F}}$ are residual matrices, and $F$ is the number of iterations (factors). The PLS approach is particularly well suited for high dimensional data due to its efficient dimensional reduction technique.

Multi-way Partial Least Squares (NPLS) regression is an algorithm in the PLS-family that is adopted to the case of tensor variables^32^. Tensors (multi-way arrays) are higher-order generalization of vectors and matrix data representation: vectors are tensors of order one, and the matrix is a second order tensor. In this paper, a tensor is denoted as $\underline{{\bf{X}}}\in {{\mathbb{R}}}^{{I}_{1}\times \ldots \times {I}_{n}}$, where $n$ represents the order of the tensor. Detailed information about the tensors is described^57^. NPLS combines the robustness of PLS regression with the ability to preserve the structure of the data, which is lost in vector-oriented approaches. For independent and dependent tensors of observation $\underline{{\bf{X}}}\in {{\mathbb{R}}}^{N\times {I}_{1}\times \ldots \times {I}_{n}}$ and $\underline{{\bf{Y}}}\in {{\mathbb{R}}}^{N\times {J}_{1}\times \ldots \times {J}_{m}}$, the NPLS iteratively constructs the linear relationship by projecting them to the space of latent variables similar to the PLS way: $\underline{{\bf{X}}}=\sum _{f=1}^{F}{{\bf{t}}}_{f}\circ {{\bf{w}}}_{f}^{1}\circ \ldots \circ {{\bf{w}}}_{f}^{n}+\underline{{\bf{E}}}$, $\underline{{\bf{Y}}}=\sum _{f=1}^{F}{{\bf{u}}}_{f}\circ {{\bf{q}}}_{f}^{1}\circ \ldots \circ {{\bf{q}}}_{f}^{m}+\underline{{\bf{F}}}$, ${{\bf{u}}}_{f}={{\bf{T}}}_{f}{{\bf{b}}}_{f}$. Here, the operator “$\circ$” is the outer product^57^, ${{\bf{T}}}_{f}=[{{\bf{t}}}_{1},\ldots ,{{\bf{t}}}_{f}]\in {{\mathbb{R}}}^{N\times f}$ and ${{\bf{U}}}_{f}=[{{\bf{u}}}_{1},\ldots ,{{\bf{u}}}_{f}]\in {{\mathbb{R}}}^{N\times f}$ are the matrices of the latent variables after $f=1,\ldots ,F$ iterations, ${{\bf{w}}}_{f}^{i}\in {{\mathbb{R}}}^{{I}_{i}}$ and ${{\bf{q}}}_{f}^{j}\in {{\mathbb{R}}}^{{J}_{j}}$ are the projection vectors, ${{\bf{b}}}_{f}$ is the vector of the linear coefficients, and $\underline{{\bf{E}}}$ and $\underline{{\bf{F}}}$ are the residual tensors.

### Recursive PLS regression

Several recursive PLS-based algorithms were proposed to treat online data flows without complete recalibration of the model^35,49,50,58–60^. Helland *et al*.^49^ presented the Recursive PLS algorithm, where the old data from the matrices ${{\bf{X}}}_{{t}_{0}}\in {{\mathbb{R}}}^{{N}_{0}\times n}$ and ${{\bf{Y}}}_{{t}_{0}}\in {{\mathbb{R}}}^{{N}_{0}\times m}$, collected before the moment ${t}_{0}$, are captured by their loading matrices ${{\bf{P}}}_{{t}_{0}}^{T}\in {{\mathbb{R}}}^{F\times n}$ and ${{\bf{Q}}}_{{t}_{0}}^{T}\in {{\mathbb{R}}}^{F\times m}$. The new matrices ${{\bf{X}}}_{{t}_{1}}\in {{\mathbb{R}}}^{{N}_{1}\times n}$ and ${{\bf{Y}}}_{{t}_{1}}\in {{\mathbb{R}}}^{{N}_{1}\times m}$ are concatenated to ${{\bf{P}}}_{{t}_{0}}^{T}$ and ${{\bf{Q}}}_{{t}_{0}}^{T}$, respectively. Then, the PLS model is recalibrated, and the new loading matrices ${{\bf{P}}}_{{t}_{1}}^{T}\in {{\mathbb{R}}}^{F\times n}$ and ${{\bf{Q}}}_{{t}_{1}}^{T}\in {{\mathbb{R}}}^{F\times m}$ are prepared for use in the next iteration of the algorithm. Thus, the method always keeps the size of the analyzed data by packing the data into the loading matrices ${\bf{P}}$ and ${\bf{Q}}$ of constant size. The main shortcoming of the RPLS consists in the possible loss of information if all of the data are not retained in the matrix of latent variables. Thus, appropriate choice of the number of factors $F$ is of crucial importance.

Qin^50^ proposed an algorithm where the hyper-parameter $F$ is estimated by the cross-validation procedure applied for the blocks of data: the whole data are divided on the blocks followed by leave-one-block-out cross-validation. However, application of the cross-validation procedure requires storing in memory the entire data set. Moreover the cross-validation procedure is not possible for online tasks. In addition, the hyper-parameters could be non-stationary and time-varying for some tasks. Despite this, if the value of the hyper-parameter $F$ is predefined in some way, then the Recursive PLS method is widely used due to relatively small memory requirement. Forgetting factor^59^, nonlinearity^50^, as well as neural networks^60^ were coupled with the Recursive PLS to model time-varying process. Recursive N-way PLS (RNPLS)— a generalization of the RPLS algorithm to the case of the tensor-input/tensor-output variables—was proposed in^35^.

Contrary to Qin’s approach^50^, Dayal and MacGregort^51,52^ proposed Recursive Exponentially Weighted PLS regression, which allows online treatment of data flow without loss of information. The algorithm is extremely fast in comparison with other recursive PLS-based approaches and is of great importance for real-time applications. Moreover, the integrating forgetting factor allows one to exponentially discount past data.

Unlike Qin’s RPLS, in the REW-PLS, the old data from the matrices ${{\bf{X}}}_{{t}_{0}}\in {{\mathbb{R}}}^{{N}_{0}\times n}$ and ${{\bf{Y}}}_{{t}_{0}}\in {{\mathbb{R}}}^{{N}_{0}\times m}$ were captured by the matrices ${{\bf{C}}}_{{\bf{X}}{\bf{X}}}^{{t}_{0}}={{\bf{X}}}_{{t}_{0}}^{T}{{\bf{X}}}_{{t}_{0}}\in {{\mathbb{R}}}^{n\times n}$ and ${{\bf{C}}}_{{\bf{X}}{\bf{Y}}}^{{t}_{0}}={{\bf{X}}}_{{t}_{0}}^{T}{{\bf{Y}}}_{{t}_{0}}\in {{\mathbb{R}}}^{m\times m}$, which are used for model calibration. When the new data is available, the covariance matrices are updated: ${{\bf{C}}}_{{\bf{X}}{\bf{X}}}^{{t}_{1}}=\lambda ({{\bf{C}}}_{{\bf{X}}{\bf{X}}}^{{t}_{0}})+{{\bf{X}}}_{{t}_{1}}^{T}{{\bf{X}}}_{{t}_{1}}$ and ${{\bf{C}}}_{{\bf{X}}{\bf{Y}}}^{{t}_{1}}=\lambda ({{\bf{C}}}_{{\bf{X}}{\bf{Y}}}^{{t}_{0}})+{{\bf{X}}}_{{t}_{1}}^{T}{{\bf{Y}}}_{{t}_{1}}$. Here, $\lambda$ $(0\le \lambda \le 1)$ is a forgetting factor. In the case of $\lambda =1$ past data cannot be discounted. There is no loss of information in the method because the calibrated model is completely identified by the covariance matrices. The detailed description of the fast model calibration algorithm based on the covariance matrices is reported^52^. However, as for all the PLS-family methods, the hyper-parameter $F$ should be predefined for the REW-PLS algorithm. The question of the efficient choice of $F$—especially for online applications—was not considered by the authors of the REW-PLS algorithm.

### Recursive Exponentially Weighted NPLS

The Recursive Exponentially Weighted NPLS is a generalization of the REW-PLS to the tensor data. On the first step, the REW-NPLS algorithm receives the tensors of observation $\underline{{\bf{X}}}\in {{\mathbb{R}}}^{N\times {I}_{1}\times \ldots \times {I}_{n}}$ and $\underline{{\bf{Y}}}\in {{\mathbb{R}}}^{N\times {J}_{1}\times \ldots \times {J}_{m}}$. Similar to the REW-PLS approach, the covariance tensors, denoted as: ${\underline{{\bf{C}}}}_{{\bf{X}}{\bf{X}}}=\underline{{\bf{X}}}{\times }_{1}\underline{{\bf{X}}}\in {{\mathbb{R}}}^{({I}_{1}\times \ldots \times {I}_{n})\times ({I}_{1}\times \ldots \times {I}_{n})}$ and ${\underline{{\bf{C}}}}_{{\bf{X}}{\bf{Y}}}=\underline{{\bf{X}}}{\times }_{1}\underline{{\bf{Y}}}\in {{\mathbb{R}}}^{({I}_{1}\times \ldots \times {I}_{n})\times ({J}_{1}\times \ldots \times {J}_{m})}$ are estimated. Here, the k-mode tensor product is denoted as “${\times }_{k}$”^57^. Similar to the NPLS method, the covariance tensors are used in the PARAFAC-based tensor decomposition procedure^32^ to estimate a set of projectors ${\{{{\bf{w}}}_{f}^{1}\in {{\mathbb{R}}}^{{I}_{1}},\ldots ,{{\bf{w}}}_{f}^{n}\in {{\mathbb{R}}}^{{I}_{n}}\}}_{f=1}^{F}$ as well as the tensor of the prediction coefficients $\underline{{\bf{B}}}=({b}_{{i}_{1},\ldots ,{i}_{n},{j}_{1},\ldots ,{j}_{m}})\in {{\mathbb{R}}}^{({I}_{1}\times \ldots \times {I}_{n})\times ({J}_{1}\times \ldots \times {J}_{m})}$; here, $F$ is a hyper-parameter representing the number of factors.

The application of the model to the new explanatory variables tensor ${\underline{{\bf{X}}}}^{{\rm{Test}}}\in {{\mathbb{R}}}^{{N}_{{\rm{Test}}}\times {I}_{1}\times \ldots \times {I}_{n}}$ for prediction of the response variables tensor ${\underline{\hat{{\bf{Y}}}}}^{{\rm{Test}}}\in {{\mathbb{R}}}^{{N}_{{\rm{Test}}}\times {J}_{1}\times \ldots \times {J}_{m}}$ was done similarly to the NPLS approach:

1 $${\underline{\hat{{\bf{Y}}}}}_{l,{j}_{1},\ldots ,{j}_{m}}^{{\rm{Test}}}=\sum _{{i}_{1},\ldots ,{i}_{n}}{\underline{{\bf{X}}}}_{l,{i}_{1},\ldots ,{i}_{n}}^{{\rm{Test}}}{\underline{{\bf{B}}}}_{{i}_{1},\ldots ,{i}_{n},{j}_{1},\ldots ,{j}_{m}},l=1,\ldots ,{N}_{{\rm{Test}}}.$$

For simplicity, we will denote it as ${\underline{\hat{{\bf{Y}}}}}^{{\rm{Test}}}={\underline{{\bf{X}}}}^{{\rm{Test}}}\underline{{\bf{B}}}$.

To update the previously calibrated model, represented by the set of ${\underline{{\bf{C}}}}_{{\bf{X}}{\bf{X}}}$, ${\underline{{\bf{C}}}}_{{\bf{X}}{\bf{Y}}}$, ${\{{{\bf{w}}}_{f}^{1},\ldots ,{{\bf{w}}}_{f}^{n}\}}_{f=1}^{F}$, and ${\bf{B}}$, with the newly available data batch ${\underline{{\bf{X}}}}^{{\rm{New}}}\in {{\mathbb{R}}}^{L\times {I}_{1}\times \ldots \times {I}_{n}}$ and ${\underline{{\bf{Y}}}}^{{\rm{New}}}\in {{\mathbb{R}}}^{L\times {J}_{1}\times \ldots \times {J}_{m}}$, and the forgetting factor $\lambda$ $(0\le \lambda \le 1)$, the covariance tensors are recalculated as:

2 $${\underline{{\bf{C}}}}_{{\bf{X}}{\bf{X}}}^{{\rm{New}}}=\lambda {\underline{{\bf{C}}}}_{{\bf{X}}{\bf{X}}}+{\underline{{\bf{X}}}}^{{\rm{New}}}{\times }_{1}{\underline{{\bf{X}}}}^{{\rm{New}}},$$

3 $${\underline{{\bf{C}}}}_{{\bf{X}}{\bf{Y}}}^{{\rm{New}}}=\lambda {\underline{{\bf{C}}}}_{{\bf{X}}{\bf{Y}}}+{\underline{{\bf{X}}}}^{{\rm{New}}}{\times }_{1}{\underline{{\bf{Y}}}}^{{\rm{New}}}.$$

**Figure 1:**
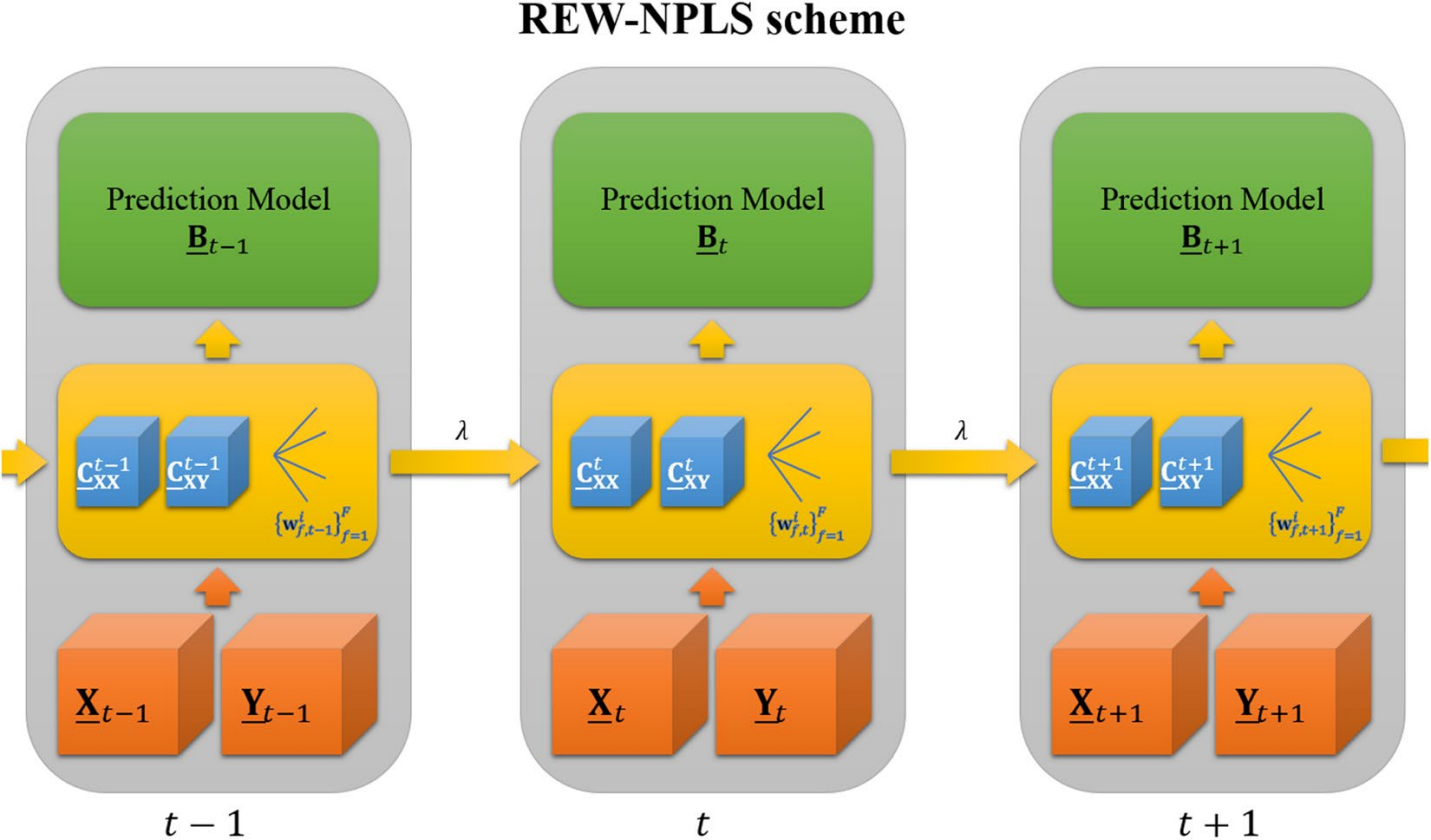
The REW-NPLS scheme. When the new tensors of observation $\{{\underline{{\bf{X}}}}_{{\boldsymbol{t}}},{\underline{{\bf{Y}}}}_{{\boldsymbol{t}}}\}$ are available, their details are then combined with the previously captured covariance tensors $\{{\underline{{\bf{C}}}}_{{\bf{X}}{\bf{X}}}^{{\boldsymbol{t}}-1},{\underline{{\bf{C}}}}_{{\bf{X}}{\bf{Y}}}^{{\boldsymbol{t}}-1}\}$, and the set of projection vectors ${\{{{\bf{w}}}_{{\boldsymbol{f}},{\boldsymbol{t}}-1}^{1},\ldots ,{{\bf{w}}}_{{\boldsymbol{f}},{\boldsymbol{t}}-1}^{{\boldsymbol{n}}}\}}_{{\boldsymbol{f}}=1}^{{\boldsymbol{F}}}$ with forgetting factor ${\lambda }$. Then the prediction model, represented by the regression coefficients ${\underline{{\bf{B}}}}_{{\boldsymbol{t}}}$, is generated.

The new projectors ${\{{{\bf{w}}}_{f}^{1,{\rm{New}}},\ldots ,{{\bf{w}}}_{f}^{n,{\rm{New}}}\}}_{f=1}^{F}$ are derived from the new covariance tensors by the PARAFAC-based tensor decomposition procedure with the previous projectors ${\{{{\bf{w}}}_{f}^{1},\ldots ,{{\bf{w}}}_{f}^{n}\}}_{f=1}^{F}$ as an initial approximation. The ${\underline{{\bf{B}}}}^{{\rm{New}}}$ is then defined. A graphical representation of the REW-NPLS method is shown in Fig. 1. A detailed description of the algorithm is given in Appendix A.

The mean-centering and rescaling of each column of the observation data should be done as in the PLS-family methods. In a time-varying process, the centering and rescaling parameters could change with time and should be continuously updated. The procedure for the matrix case is reported^51^. The same approach could be applied for the tensors because all parameters are estimated independently for each coordinate.

At moment $t$, tensors of observation ${\underline{{\bf{X}}}}_{t-1}\in {{\mathbb{R}}}^{{N}_{t-1}\times {I}_{1}\times \ldots \times {I}_{n}}$ and ${\underline{{\bf{Y}}}}_{t-1}\in {{\mathbb{R}}}^{{N}_{t-1}\times {J}_{1}\times \ldots \times {J}_{m}}$ are updated with newly available tensors ${\underline{{\bf{X}}}}_{t}\in {{\mathbb{R}}}^{{N}_{t}\times {I}_{1}\times \ldots \times {I}_{n}}$ and ${\underline{{\bf{Y}}}}_{t}\in {{\mathbb{R}}}^{{N}_{t}\times {J}_{1}\times \ldots \times {J}_{m}}$. Taking into account the forgetting factor $\lambda$, the effective number of observation at $t$ is ${N}^{{\rm{eff}}(t)}=\lambda {N}_{t-1}+{N}_{t}=\lambda {N}^{{\rm{eff}}(t-1)}+{N}_{t}$.

The sum and the sum of squares of the elements along the observation modality are estimated as:

4 $${\underline{{\bf{S}}}}^{{\rm{eff}}(t)}=\lambda {\underline{{\bf{X}}}}_{t-1}{\times }_{1}{1}^{{N}_{t-1}}+{\underline{{\bf{X}}}}_{t}{\times }_{1}{1}^{{N}_{t}}\in {{\mathbb{R}}}^{{I}_{1}\times \ldots \times {I}_{n}},$$

5 $${\underline{{\bf{S}}{\bf{S}}}}^{{\rm{eff}}(t)}=\lambda {{\underline{{\bf{X}}}}_{t-1}}^{\ast 2}{\times }_{1}{1}^{{N}_{t-1}}+{{\underline{{\bf{X}}}}_{t}}^{\ast 2}{\times }_{1}{1}^{{N}_{t}}\in {{\mathbb{R}}}^{{I}_{1}\times \ldots \times {I}_{n}},$$

where “${\underline{{\bf{X}}}}^{\ast 2}$” represents the element-wise square of the tensor $\underline{{\bf{X}}}$. The effective mean and the standard deviation along the first modality of the tensors at time $t$ are:

6 $${\underline{{{\boldsymbol{\mu }}}_{1}{\bf{X}}}}^{{\rm{eff}}(t)}={\underline{{\bf{S}}}}^{{\rm{eff}}(t)}/{N}^{{\rm{eff}}(t)}\in {{\mathbb{R}}}^{{I}_{1}\times \ldots \times {I}_{n}},$$

7 $${\underline{{{\boldsymbol{\sigma }}}_{1}{\bf{X}}}}^{{\rm{eff}}(t)}=\sqrt{\frac{{\underline{{\bf{S}}{\bf{S}}}}^{{\rm{eff}}(t)}-{({\underline{{\bf{S}}}}^{{\rm{eff}}(t)})}^{\ast 2}/{N}^{{\rm{eff}}(t)}}{{N}^{{\rm{eff}}(t)}-1}}\in {{\mathbb{R}}}^{{I}_{1}\times \ldots \times {I}_{n}}.$$

For simplicity of notation, we omit the indexes and write $\underline{{\boldsymbol{\mu }}{\bf{X}}}$ and $\underline{{\boldsymbol{\sigma }}{\bf{X}}}$. The tensors $\underline{{\boldsymbol{\mu }}{\bf{Y}}}\in {{\mathbb{R}}}^{{J}_{1}\times \ldots \times {J}_{m}}$ and $\underline{{\boldsymbol{\sigma }}{\bf{Y}}}\in {{\mathbb{R}}}^{{J}_{1}\times \ldots \times {J}_{m}}$ are identified similarly.

To apply the centered model to the non-centered testing data ${\underline{{\bf{X}}}}^{{\rm{Test}}}$, equation (1) should be rewritten:

8 $${\underline{\hat{{\bf{Y}}}}}^{{\rm{Test}}}={\underline{{\bf{X}}}}^{{\rm{Test}}}\tilde{\underline{{\bf{B}}}}+{\underline{{\bf{Y}}}}_{0},$$

where $$\tilde{\underline{{\bf{B}}}}=({b}_{{i}_{1},\ldots ,{i}_{n},{j}_{1},\ldots ,{j}_{m}}{\underline{{\boldsymbol{\sigma }}{\bf{Y}}}}_{{j}_{1},\ldots ,{j}_{m}}/{\underline{{\boldsymbol{\sigma }}{\bf{X}}}}_{{i}_{1},\ldots ,{i}_{n}})=\underline{{\bf{B}}}\underline{{\boldsymbol{\sigma }}{\bf{Y}}}/\underline{{\boldsymbol{\sigma }}{\bf{X}}}$$, $${\underline{{\bf{Y}}}}_{0}=\underline{{\boldsymbol{\mu }}{\bf{Y}}}-\underline{{\boldsymbol{\mu }}{\bf{X}}}\tilde{\underline{{\bf{B}}}}$$.

A detailed description of the normalization procedure is given in Appendix B.

Determination of the appropriate number of factor $F$ is a common task for all PLS-family methods. While the cross-validation approach^55^ is widely used for this purpose, it cannot be efficiently applied for online modeling. Here, we propose a procedure for recursive estimation of the optimal number of factors (${F}^{\ast }$) for REW-NPLS algorithm. The main idea exploits the newly available data as a testing set for the currently available models before the models are updated.

At time $t$, the new tensors of observation ${\underline{{\bf{X}}}}_{t}$ and ${\underline{{\bf{Y}}}}_{t}$ are available. The current prediction model ${\underline{{\bf{B}}}}_{t-1}^{{F}_{{\rm{\max }}}}$ resulted from the previous data and is computed for the maximum considered number of factors ${F}_{{\rm{\max }}}$. The distinctive property of the REW-NPLS algorithm consists in the ability to generate all intermediate models $\{{\underline{{\bf{B}}}}^{1},\ldots ,\,{\underline{{\bf{B}}}}^{{F}_{{\rm{\max }}}-1}\}$ during the computation of ${\underline{{\bf{B}}}}^{{F}_{{\rm{\max }}}}$, see Appendix A. All models defined in the previous step $\{{\underline{{\bf{B}}}}_{t-1}^{1},\ldots ,\,{\underline{{\bf{B}}}}_{t-1}^{{F}_{{\rm{\max }}}}\}$ could be applied to the newly available tensor of observation ${\underline{{\bf{X}}}}_{t}$ that results in a set of predictions $\{{\widehat{\underline{{\bf{Y}}}}}_{t}^{1},\ldots ,{\widehat{\underline{{\bf{Y}}}}}_{t}^{{F}_{{\rm{\max }}}}\}$. The error of each prediction could be estimated: $\{{e}_{t}^{1},\ldots ,{e}_{t}^{{F}_{{\rm{\max }}}}\}$, where ${e}_{t}^{f}=\gamma {e}_{t-1}^{f}+{\rm{ERROR}}({\widehat{\underline{{\bf{Y}}}}}_{t}^{f},{\underline{{\bf{Y}}}}_{t})$, with the forgetting factor $\gamma \,(0\le \gamma \le 1)$. The optimal number of factors, corresponding to the moment $t$ is defined as

9 $${F}_{t}^{\ast }={{\rm{argmin}}}_{f}{\{{e}_{t}^{f}\}}_{f=1}^{{F}_{{\rm{\max }}}},$$

and the optimal model is

10 $${\underline{{\bf{B}}}}_{t}^{\ast }={\underline{{\bf{B}}}}_{t}^{{F}_{t}^{\ast }}.$$

**Figure 2:**
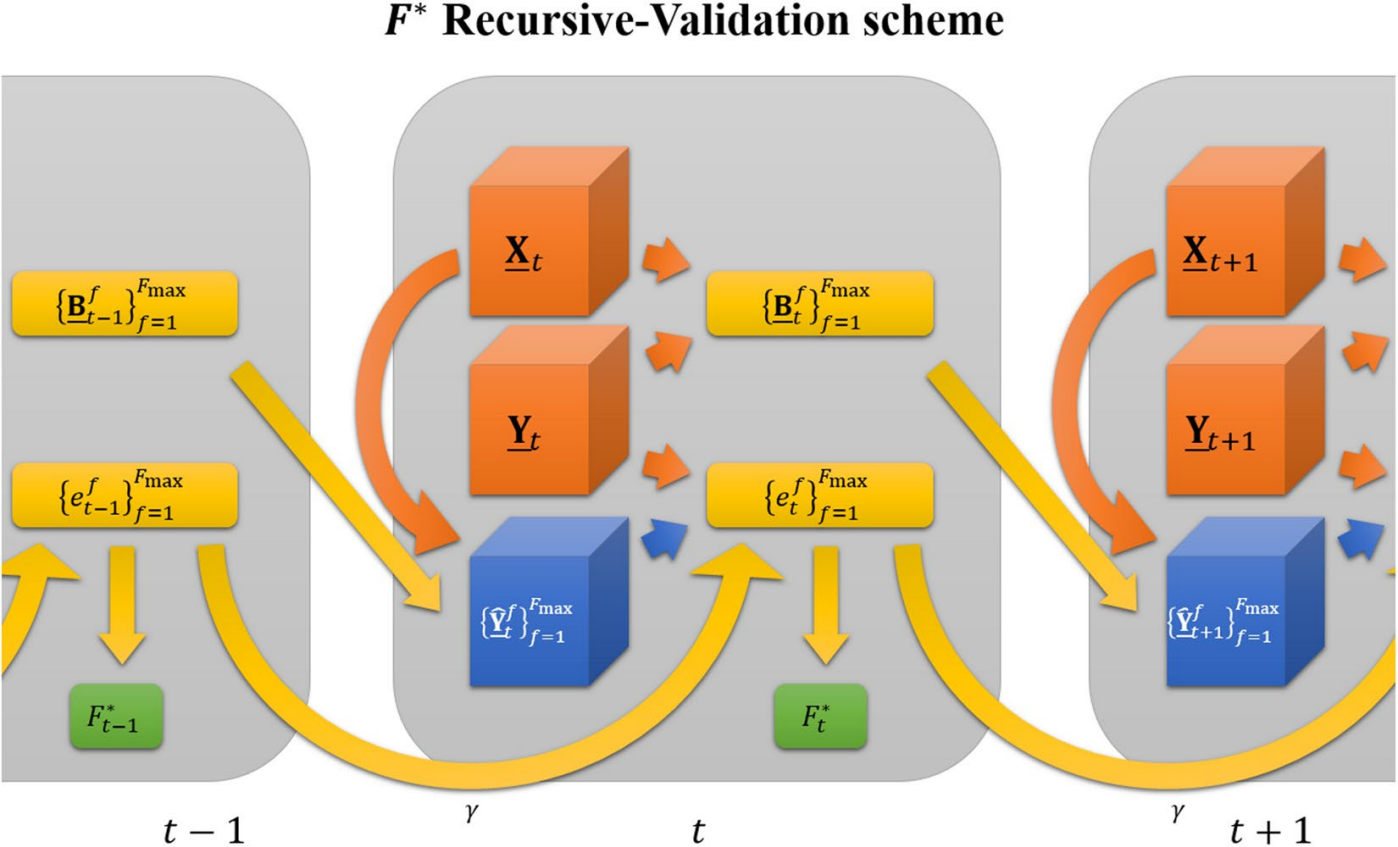
The Recursive-Validation scheme for the number of factors hyper-parameter (${F}$) identification. When the new tensors of observation $\{{\underline{{\bf{X}}}}_{{\boldsymbol{t}}},{\underline{{\bf{Y}}}}_{{\boldsymbol{t}}}\}$ are available, the previously defined models $\{{\underline{{\bf{B}}}}_{{\boldsymbol{t}}-1}^{1},\ldots ,\,{\underline{{\bf{B}}}}_{{\boldsymbol{t}}-1}^{{{\boldsymbol{F}}}_{{\bf{m}}{\bf{a}}{\bf{x}}}}\}$ are tested on the new data and resulted in $\{{\widehat{\underline{{\bf{Y}}}}}_{{\boldsymbol{t}}}^{1},\ldots ,{\widehat{\underline{{\bf{Y}}}}}_{{\boldsymbol{t}}}^{{{\boldsymbol{F}}}_{{\bf{m}}{\bf{a}}{\bf{x}}}}\}$. The prediction errors ${\{{{\boldsymbol{e}}}_{{\boldsymbol{t}}}^{{\boldsymbol{f}}}\}}_{{\boldsymbol{f}}=1}^{{{\boldsymbol{F}}}_{{\bf{m}}{\bf{a}}{\bf{x}}}}$ are estimated considering the previous errors with the forgetting factor ${\boldsymbol{\gamma }}$. The number of factors ${{F}}_{{\boldsymbol{t}}}^{\ast }={{\rm{a}}{\rm{r}}{\rm{g}}{\rm{m}}{\rm{i}}{\rm{n}}}_{{\boldsymbol{f}}}{\{{{\boldsymbol{e}}}_{{\boldsymbol{t}}}^{{\boldsymbol{f}}}\}}_{{\boldsymbol{f}}=1}^{{{\boldsymbol{F}}}_{{\bf{m}}{\bf{a}}{\bf{x}}}}$ correspond to the minimal error at the current moment *t*; this is considered to be optimal for the current moment.

The detailed description of the Recursive-Validation procedure is given in Appendix C. Its graphic representation is shown in Fig. 2.

### Experiments in Monkeys

#### Data description

**Figure 3:**
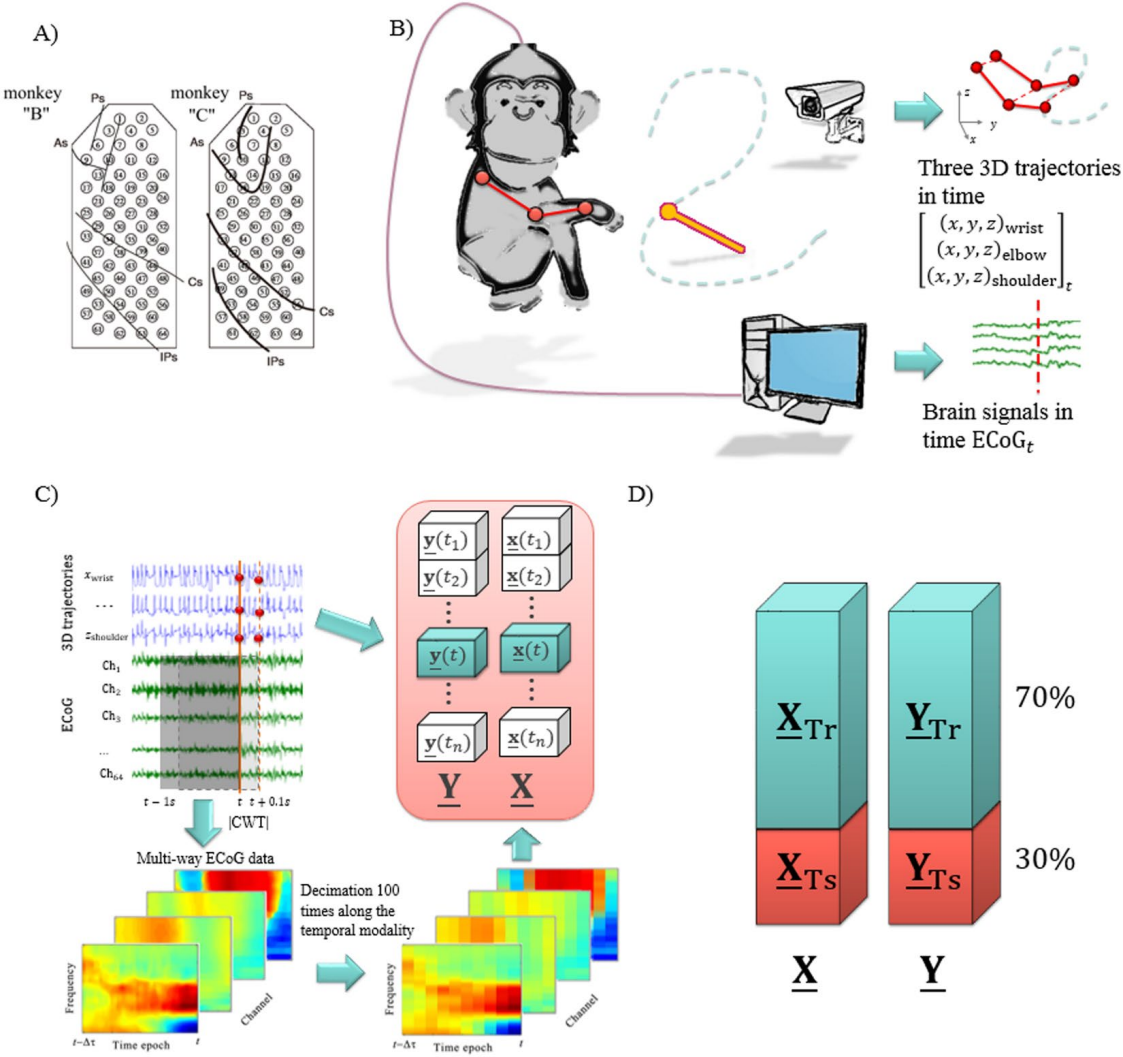
ECoG-based primate experiments. (**A**) 64 electrodes were implanted in the epidural space of the left hemisphere of two Japanese macaques denoted as monkey “B” and “C”. Ps: principal sulcus; As: arcuate sulcus; Cs: central sulcus; IPs: intraparietal sulcus^11^. (**B**) The scheme of the experiment. The monkey is following the food with its right hand. Monkey’s ECoG activity is recorded simultaneously with 3D coordinates of the right wrist, elbow, and shoulder. (**C**) For each time ${\boldsymbol{t}}$, to form the explanatory variable $\underline{{\bf{x}}}(t)\in {{\mathbb{R}}}^{15\times 10\times 64}$, the ECoG signal from 64 channels is mapped by the Continuous Wavelet Transform with 15 frequencies (10, 20, …, 150 Hz). Then, the absolute values of the wavelet coefficients are decimated 100 times along the temporal modality, i.e., 1000 points, representing 1 second; these are decimated to 10 points. The response variable $\underline{{\bf{y}}}({t})\in {{\mathbb{R}}}^{3\times 3}$ is formed from the corresponding 3D coordinates (${x},{y},{z}$) of monkey’s wrist, elbow, and shoulder. The next epoch was taken with a time step of 100 ms. (**D**) Data tensors are split into non-overlapping training (70%) and test (30%) sub-tensors.

To validate the proposed approaches, the publicly available dataset is considered (http://neurotycho.org/epidural-ecog-food-tracking-task). The data are recorded and distributed by the Laboratory for Adaptive Intelligence, BSI, RIKEN. All procedures were performed in accordance with protocols approved by the RIKEN ethics committee. The experiments consisted in recording of epidural ECoG signals simultaneously with continuous 3D trajectories of a Japanese macaque’s right wrist, elbow, and shoulder^25,61^. The experiments were carried out in two monkeys denoted as “B” and “C”. Overall, 20 recordings (10 for each monkey) are provided^11^. The length of each recording was approximately 15 minutes. To record the hand motions (shoulders, elbows, and wrists), an optical motion capture system (Vicon Motion System, Oxford, UK) with a sampling rate of 120 Hz was applied. The ECoG signals were recorded from 64 electrodes (Blackrock Microsystems, Salt Lake City, UT, USA) with a sampling rate of 1 kHz, implanted in the epidural space of the left hemisphere (Fig. 3A). Each experiment showed the monkeys pieces of food, and they were trained to receive it with the right hands. The location of the food was random at a distance of about 20 cm from the monkeys. A scheme of the experiment is shown in Fig. 3B.

#### Feature extraction and tensors formation

An input data feature tensor $\underline{{\bf{X}}}$ was formed from the ECoG epochs containing 1 second of the signal taken continuously with a time step of 100 ms. Each ECoG epoch was mapped to the spatial-temporal-frequency space by continuous wavelet transform (CWT). The complex Morlet wavelet was chosen as a mother wavelet^25,38,62,63^. The frequency band from 10 to 150 Hz with step 10 Hz was chosen^11^. Absolute values of the wavelet coefficients were decimated along the temporal modality 100 times by averaging 10 sliding windows each 100 ms long. A detailed description of the feature extraction procedure has been reported^24^. The tensor of the output variables $\underline{{\bf{Y}}}$ was formed from the corresponding 3D coordinates of the monkey’s right wrist, elbow, and shoulder. Figure 3C represents the scheme of the data preparation procedure.

According to^63^, each 15-minute recording from the available dataset of 20 files was split into non-overlapping training (~10 first minutes) and test (~5 last minutes) subsets; see Fig. 3D:

11 $${\underline{{\bf{X}}}}^{{\rm{recoding}}}=[\begin{array}{c}{\underline{{\bf{X}}}}_{{\rm{training}}}^{{\rm{recoding}}}\in {{\mathbb{R}}}^{7000\times 15\times 10\times 64}\\ {\underline{{\bf{X}}}}_{{\rm{test}}}^{{\rm{recoding}}}\in {{\mathbb{R}}}^{3000\times 15\times 10\times 64}\end{array}],$$

12 $${\underline{{\bf{Y}}}}^{{\rm{recoding}}}=[\begin{array}{c}{\underline{{\bf{Y}}}}_{{\rm{training}}}^{{\rm{recoding}}}\in {{\mathbb{R}}}^{7000\times 3\times 3}\\ {\underline{{\bf{Y}}}}_{{\rm{test}}}^{{\rm{recoding}}}\in {{\mathbb{R}}}^{3000\times 3\times 3}\end{array}].$$

The models provided by the different methods are calibrated on the training tensors and validated on the corresponding test tensors.

### Experiments in humans

#### Data description

**Figure 4:**
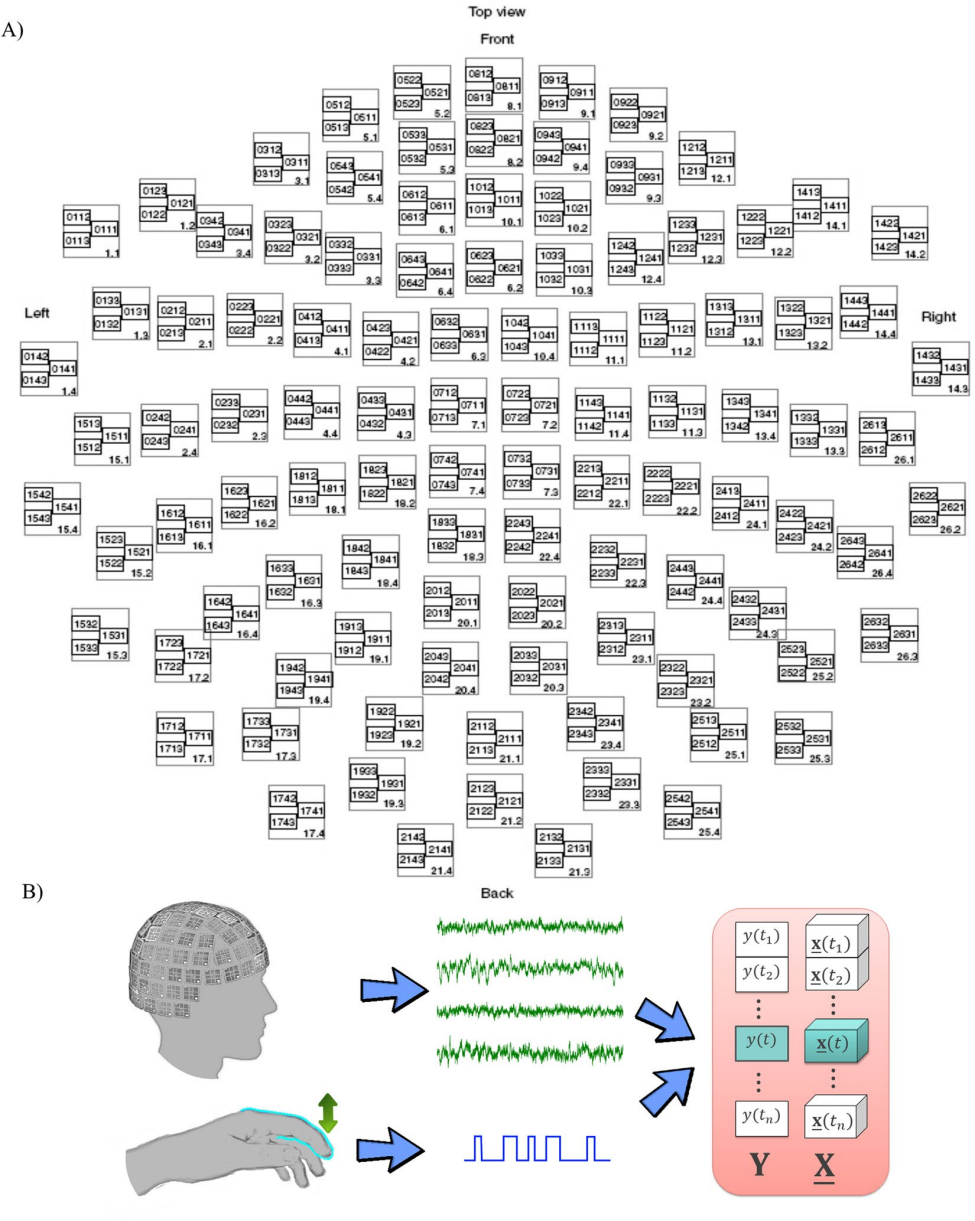
MEG-based experiments in human. (**A**) Elekta Neuromag electrodes scheme. (**B**) The scheme of the experiment. The subject voluntarily performs up/down movements of the index finger. For each time moment *t*, to form the explanatory variable $\underline{{\bf{x}}}({t})\in {{\mathbb{R}}}^{20\times 10\times 306}$, the MEG signal from 306 channels is mapped by the Continuous Wavelet Transform with 20 frequencies (5, 10, …, 100 Hz). Then, the absolute values of the wavelet coefficients are decimated 100 times along the temporal modality. The response variable ${\boldsymbol{y}}({t})\in \{0,1\}$ is formed from the corresponded down/up position of the index finger at the moment *t*. The next epoch was taken with a time step of 100 ms.

The experiments were carried out by the Clinatec team (CEA-LETI-CLINATEC^®^, Grenoble, France) and recorded the MEG signals in four healthy subjects (25–43 years old) with no known neurological or psychiatric problem. This study was approved by Comité de protection des personnes Sud-Est V ethics committee (Clinical Trial NTC02574026, AFFSAPS 2010-A00421-38). All experiments were performed in accordance with relevant guidelines and regulations. Informed consent was obtained from all participants. During the experiments, each subject lifted his/her index finger without any external cue or conditional stimulus. The binary position of the finger (“up” or “down”) was registered by a laser beam. Only one finger was moved during each session. The sessions varied between 5 and 20 minutes and provided 90 to 520 self-paced finger movements. The brain activity recordings were made in a magnetically shielded room using 306-channel MEG devices (Elekta Neuromag, Helsinki, Finland). The device provides MEG data recorded from 102 triple sensor units (one magnetometer and two orthogonal planar gradiometers) with a sampling rate of 1 kHz (Fig. 4A).

#### Feature extraction and tensors formation

Similarly to the experiments in monkeys, an input data feature tensor $\underline{{\bf{X}}}$ was formed from the MEG epochs containing 1 second of the signal taken continuously with a time step of 100 ms. Each MEG epoch, was mapped to the spatial-temporal-frequency space by CWT based on the complex Morlet wavelet. The frequency band from 5 to 100 Hz with step 5 Hz was used. The binary vector of the output variables ${\bf{Y}}$ was formed from the corresponding position of the index finger. Figure 4B represents the scheme of the experiments and the data preparation procedure.

Four subjects participated in the experiments. All four sessions were recorded with each subject: 2 with left and 2 with right index movements. Overall, 16 recordings were prepared. Each recording was split on two non-overlapping training (70%) and test (30%) subsets.

### Prediction performance evaluation

To predict performance evaluation, several criteria have been applied in BCI experiments^64^. In this paper, we use Pearson correlation ($r$) between predicted and observed response because it is sufficiently informative and could be easily computed and interpreted. It is commonly used in BCIs to assess decoders^11,25,62,63^.

## Results

### Experiments in monkeys

Here we consider vector- and tensor-oriented approaches (PLS and NPLS) as well as their recursive modification (REW-PLS and REW-NPLS). For the recursive approaches, the training sets were split on the non-overlapping 10-second long batches with 100 epochs per batch and about 70 batches per training set. The recursive models (REW-PLS and REW-NPLS) were adjusted each time when the new batch arrived. The non-recursive models (PLS and NPLS) were calibrated just once on the entire training set. For the recursive approaches, a forgetting factor $\lambda =1$ was chosen, i.e., there was no discounting of past data batches.

#### Prediction accuracy and robustness

**Figure 5:**
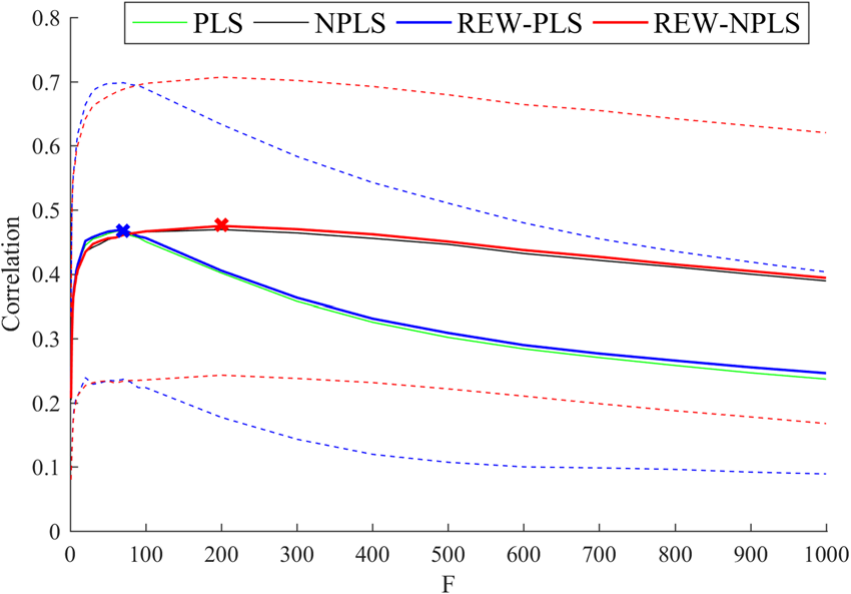
Prediction accuracy and robustness to the factors number parameter (*F*). The maximal prediction accuracy is represented by the crosses in the figure and is almost equivalent for all the approaches. However, in comparison with vector-oriented methods (PLS, REW-PLS), the tensor-based approaches (NPLS, REW-NPLS) demonstrate better robustness to variability of *F*. Dotted lines represent the standard deviation of the corresponding correlations.

The methods were compared based on their prediction accuracy and robustness to the value of the number of factors. For each approach, namely, PLS, NPLS, REW-PLS, and REW-NPLS, the correlation between predicted and observed trajectories is represented in Fig. 5 and is averaged over recordings, hand’s joints (shoulder, elbow, wrist), and coordinates. The optimal values of the factor numbers that provide the maximum of averaged correlations and the corresponding correlations are given in Table 1.

**Table 1:** The optimal values of the factors number F* and the values of the corresponding correlations ${{r}}^{\ast }={r}({{F}}^{\ast })$ on the test data for the comparison methods.

**Method**	**Optimal Factors Number** (***F*** *)	**Correlation** (***r*** *)
PLS	70	0.47 ± 0.23
REW-PLS	70	0.47 ± 0.23
NPLS	200	0.47 ± 0.23
REW-NPLS	200	0.48 ± 0.23

#### Recursive-Validation of factors number

**Figure 6:**
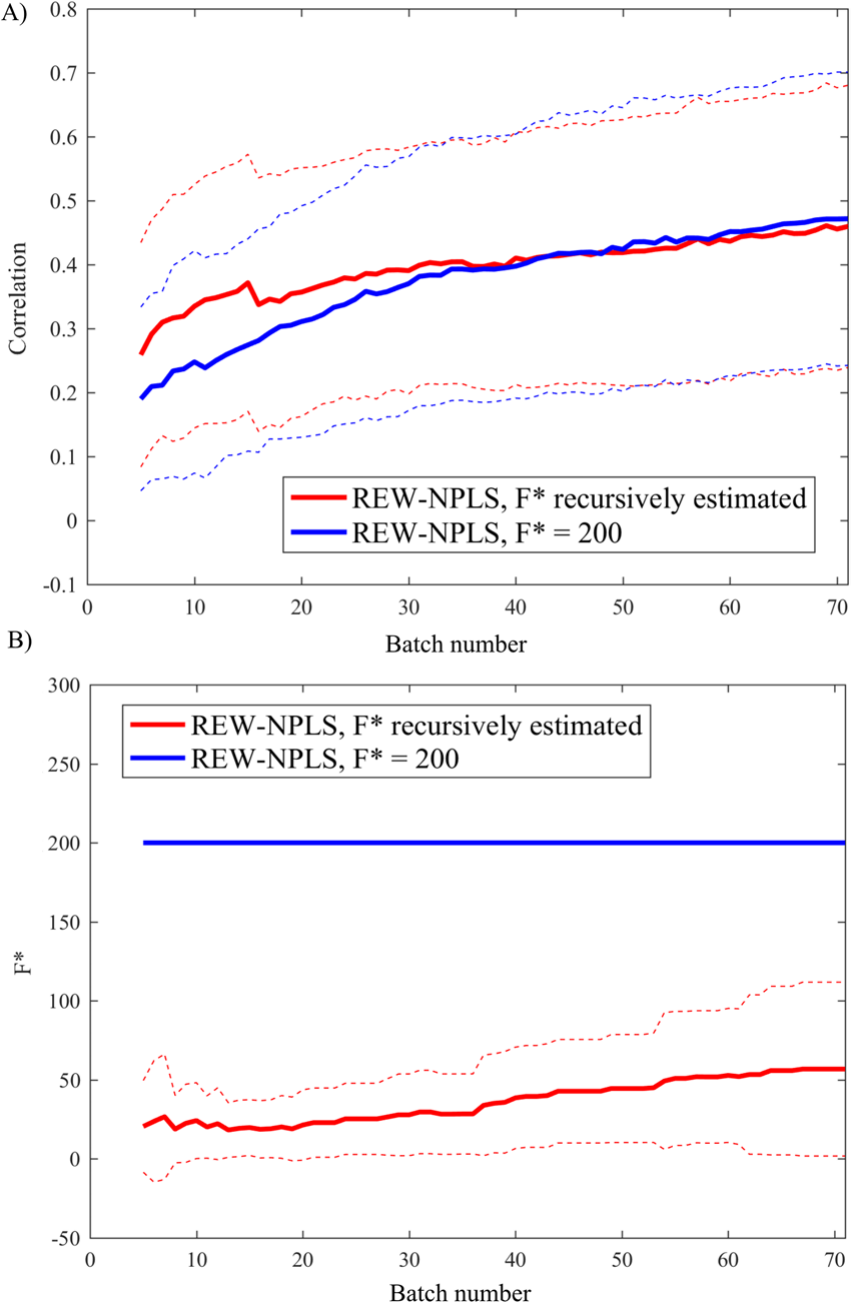
Comparison of the prediction correlation for REW-NPLS; the preliminary estimate of optimal number of factors (${F}^{\ast }=200$) vs. the Recursive-Validation of the factors number. The size of each data-batch is equal to 10 seconds (100 epochs), and 70 batches are available. The data are averaged through 20 recordings and 9 coordinates. The maximum number of factors ${{F}}_{max}=300$ is taken in the Recursive-Validation approach. (**A**) The method with Recursive-Validation of F* significantly outperforms the one with optimal ${F}^{\ast }=200$ until 25 batches are analyzed. The difference then became insignificant (ANOVA test, significance level ${\boldsymbol{\alpha }}=0.05$). (**B**) When 70 batches are treated, the dynamically estimated number of factors is ${{F}}_{{\bf{R}}{\bf{V}}}^{\ast }=57\ll {F}^{\ast }=200$, whereas the difference in prediction accuracy is insignificant. Dotted lines represent standard deviations.

To assess the quality of the Recursive-Validation procedure in estimating the number of factors $F$, the prediction results were compared to the REW-NPLS with an optimal number of factors ${F}^{\ast }=200$ (see Table 1). To model the online data flow, the training dataset was split on seventy 10-second non-overlapping batches (100 epochs per batch). When the new data-batch arrives, the model was adjusting according to the new data and was then tested on entire testing set. For the Recursive-Validation procedure, the maximal number of tested factors was ${F}_{{\rm{\max }}}=300\,$(Fig. 6A). The REW-NPLS with dynamic adaptation of $F$ significantly outperformed the REW-NPLS with an optimal value of factors ($F={F}^{\ast }$) until the number of batches reached 25. The difference became insignificant (ANOVA test, significance level α = 0.05). Figure 6B demonstrates the optimal number of factors (${F}_{{\rm{RV}}}^{\ast }$) estimated by the Recursive-Validation procedure as a function of the number of analyzed batches. When the complete training tensor was analyzed (all 70 batches), the dynamically-estimated optimal number of factors was ${F}_{{\rm{RV}}}^{\ast }=57\ll {F}^{\ast }=200$; the prediction correlations are comparable.

#### Modality influences analysis

**Figure 7:**
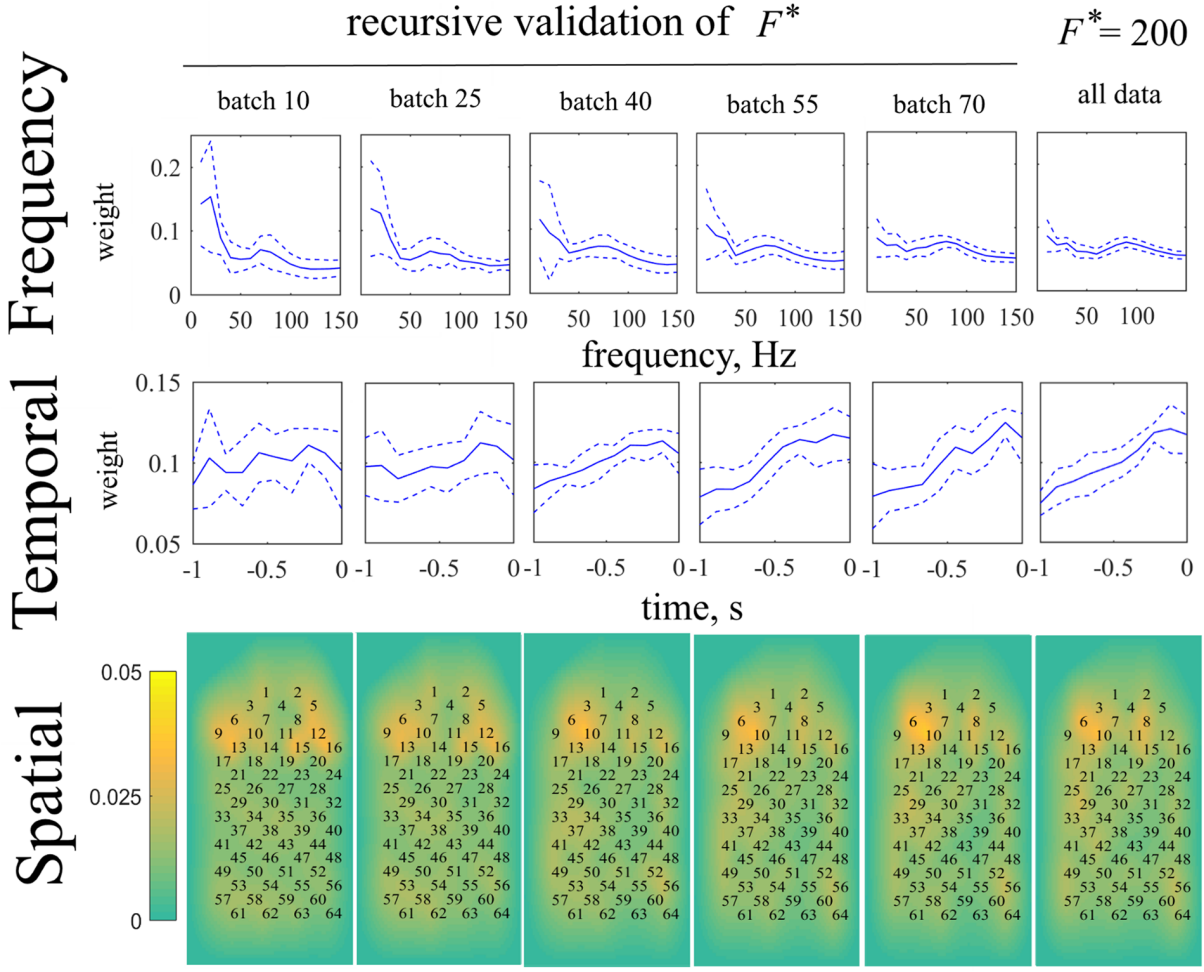
The influences on the predictive models of the elements in frequency, temporal, and spatial modalities identified by the REW-NPLS (${F}^{\ast }=200$) on the complete training set, and the REW-NPLS with Recursive-Validation of F*. The batch numbers 10, 25, 40, 55, and 70 (complete dataset) demonstrate the model’s variability over time. The modalities influences are averaged over 20 recordings and 9 coordinates. Dotted lines represent the standard deviation of the corresponding results.

Tensor-based approaches facilitate study of the resulting models in different modalities^33^. The predictive models are identified by the REW-NPLS with Recursive-Validation of ${F}^{\ast }$ and are compared to the model identified on the entire training data with an optimal number of factors ${F}^{\ast }=200$; see Fig. 7. The models are represented by their averaged influences^65^ in spatial, temporal, and frequency modalities. Each influence is computed as the weight of sum of the absolute values of the model’s coefficients along the corresponding modality. The weights are averaged over the recordings and coordinates. For the case of the Recursive-Validation of ${F}^{\ast }$, the models are shown for batch numbers 10, 25, 40, 55, and 70 (complete dataset).

### Experiments in humans

#### Recursive-Validation vs. Cross-Validation

To evaluate the proposed Recursive-Validation procedure, the number of factors estimated by this approach was compared to the number of factors estimated by the standard 10-fold cross-validation procedure. The number of factors was taken from the interval $F\in [1,\,20]$. Table 2 demonstrates the optimal number of factors ${F}^{\ast }$ estimated by the methods as well as the value of the corresponding correlation ${r}^{\ast }=r({F}^{\ast })$ on the test sets. The difference in the resulting correlations was not significant for either left- or right indexes (ANOVA test, significance level $\alpha =0.05$).

**Table 2:** The optimal values of the factors number F* and the corresponding correlations ${{r}}^{\ast }={r}({{F}}^{\ast })$ for the comparison methods. The results are averaged over 8 recordings for the left- and right fingers.

**Method**	**Finger**	**Optimal Factors Number** **(** ${{\boldsymbol{F}}}^{{\boldsymbol{\ast }}}$ *)*	**Correlation** ${\boldsymbol{(}}{{\boldsymbol{r}}}^{{\boldsymbol{\ast }}}{\boldsymbol{)}}$
10-fold Cross-Validation	Left	6 ± 3	0.26 ± 0.10
	Right	6 ± 2	0.27 ± 0.08
Recursive-Validation	Left	5 ± 2	0.28 ± 0.10
	Right	5 ± 2	0.29 ± 0.10

#### Modality influences analysis

**Figure 8:**
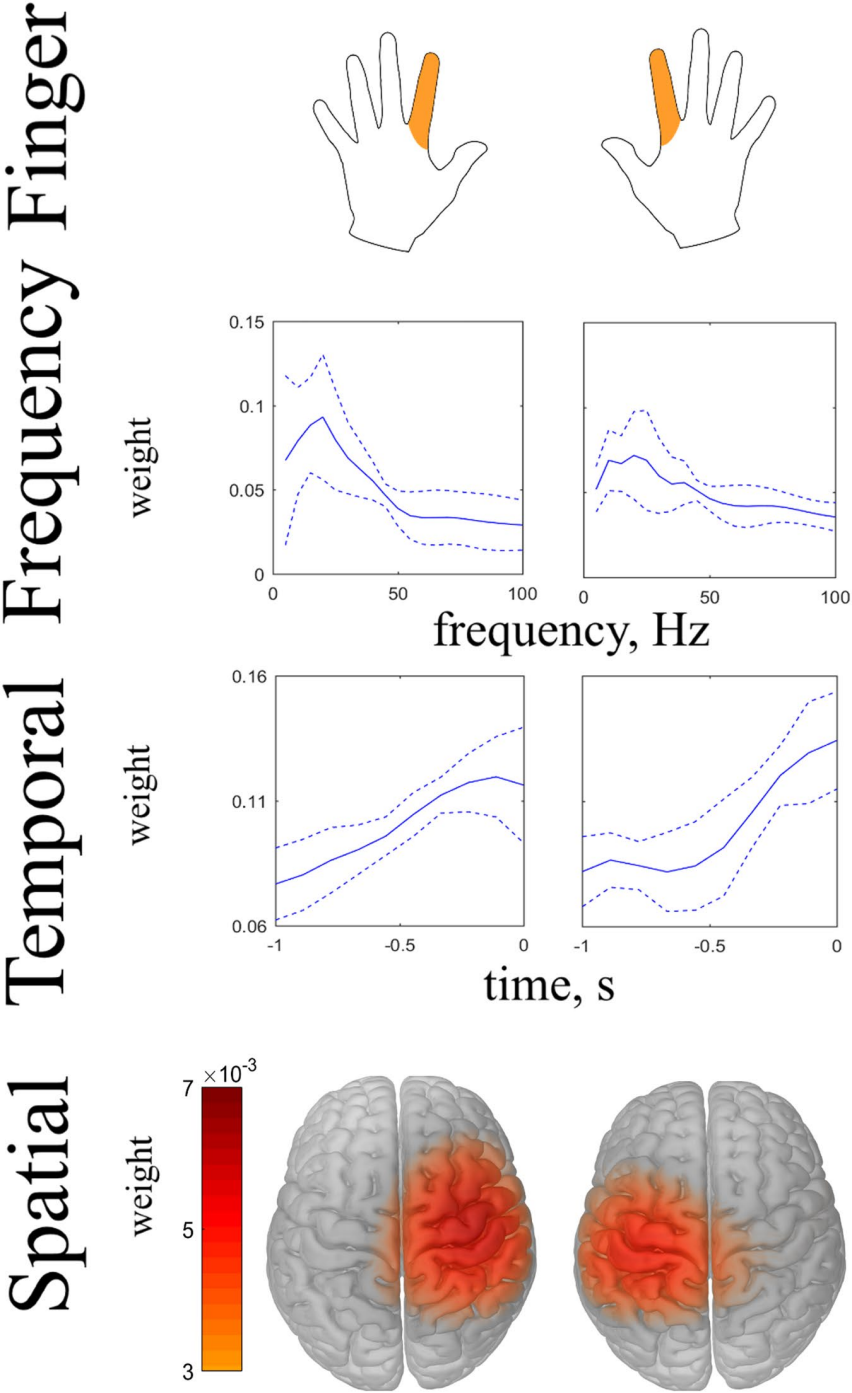
The influences on the predictive models of the elements in frequency, temporal, and spatial modalities identified by the REW-NPLS with Recursive-Validation of F* on the complete training set. The modalities influence was averaged over 8 recordings for the left- and right fingers. Dotted lines represent the standard deviation of the corresponding results.

The predictive models were identified by the REW-NPLS with Recursive-Validation of ${F}^{\ast }$ on the complete training set and are shown in Fig. 8. The models are represented by their averaged influence in frequency, temporal, and spatial modalities. The weights are averaged over 4 subjects (2 recording per subject) for left and right finger self-paced movements.

## Implementation

The crucial point for practical application of signal-processing approaches in BCI systems is a possibility for of the real-time computations. All methods proposed here are implemented in Matlab^®^ (The MathWorks, Inc., Natick, US) and can function in real-time on a standard computer (Intel Xenon E5-2620, 2 GHz, RAM 32 Gb). Due to real-time functioning requirements, blocks of applications and adaptations of the model are separated and implemented in two independent processes while communicating through shared memory. The Model Application Block provides a 10-Hz decision rate, i.e. control command is generated each time that the new 100 ms data block arrives. The Model Adaptation Block is waiting when the new data stack of 10 seconds is acquired. The model then is updated with newly available data (REW-NPLS algorithm with Recursive-Validation of the factors number).

**Figure 9:**
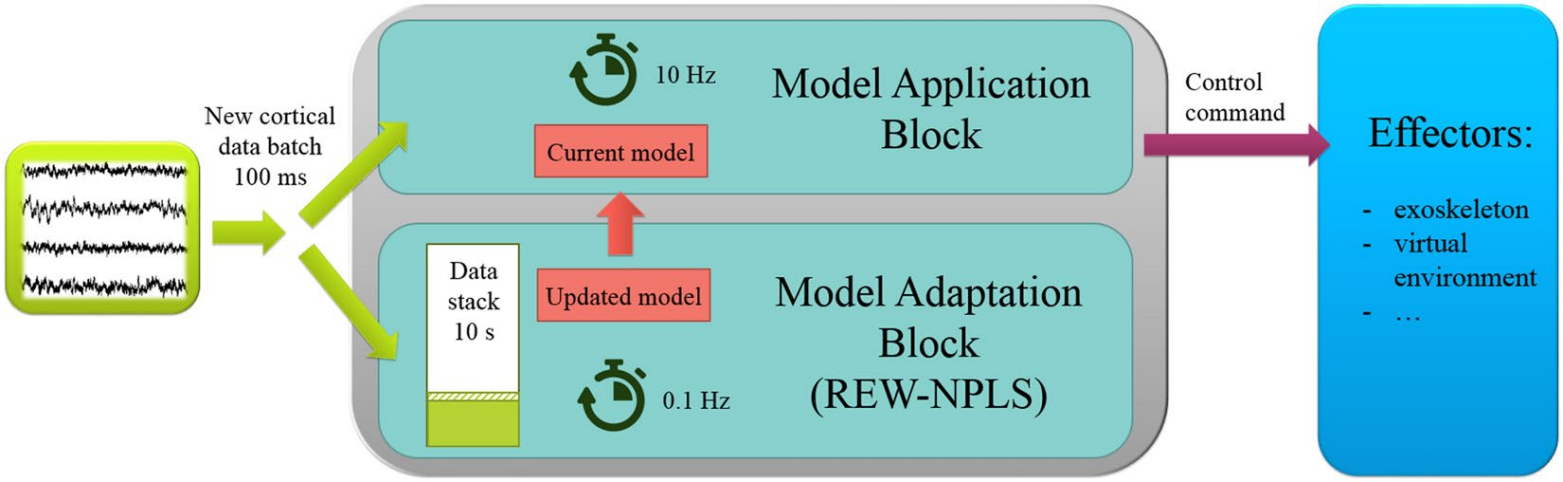
Implementation scheme of the real-time operating system. The control command for an external device is generated every 100 ms by the Model Application Block. The model is adjusted every 10 seconds by the Model Adaptation Block based on the REW-NPLS algorithm with Recursive-Validation of the factor number.

When the updated model is prepared, it replaces the current model in the Model Application Block. For the Model Application Block, the processing time for generation of a control command with a new 100 ms data block (sampling rate 1 kHz, 64 channels, 15 analyzed frequencies) is 37 ± 8 ms. Updating the model with 10 seconds of data stack in the Model Adaptation Block takes 3.41 ± 0.06 s. Thus, both blocks meet the real-time functioning requirements (37 ms ≪ 100 ms and 3.41 s ≪ 10 s). The scheme of the system is given in Fig. 9.

## Discussion

Decoding of the neural activity for BCI systems is a challenging task^1^. The huge dimension, considerable data variability in time, as well as real-time computational requirements impose essential limitations on real-life applications of BCIs^66,67^.

The paper proposes the REW-NPLS algorithm for recursive analysis of multimodal data. While the standard vector-oriented approaches applied in BCI do not consider the structure of the analyzed data^11,68^, the tensor-based methods are commonly not adjusted for online computations and require complete recalibration when the new data are available^38,69^. Previously^35^, the RNPLS algorithm allows online treatment of multi-modal data, however, only part of information is extracted from arriving data batches. This could lead to a loss in the quality of the prediction model. The REW-NPLS method unites both tensor-based structures of NPLS with the possibility of efficient online treatment of data without information loss inherited from the REW-PLS.

The crucial parameter of the PLS-family methods is the number of iterations (number of factors, $F$) used in the model for projections into the space of latent variables^70^. The robustness of the methods in the case of $F$’s suboptimal values is very important because there are mostly heuristic algorithms for selection of the optimal value of $F$. The task is especially critical for online data flows when the values of the parameters are unfixed and could vary widely with time.

In the current paper, the proposed REW-NPLS approach was studied for the robustness of its prediction accuracy to the suboptimal values of the number of factors on the ECoG data. The recursive approaches (REW-NPLS, REW-PLS) do not concede to the corresponding non-recursive approaches (NPLS, PLS) according to their prediction quality result; Fig. 5 and Table 1. Moreover, the recursive approaches are slightly better and this could be explained by the additional stability provided by the batch-wise normalization. In comparison with the tensor-based methods (NPLS, REW-NPLS), the vector-oriented approaches (PLS, REW-PLS) require fewer factors to achieve best correlation: ${F}_{{\rm{REW}}-{\rm{PLS}}}^{\ast }=70$, ${r}_{{\rm{REW}}-{\rm{PLS}}}^{\ast }=0.47\pm 0.23$, ${F}_{{\rm{REW}}-{\rm{NPLS}}}^{\ast }=200$, ${r}_{{\rm{REW}}-{\rm{NPLS}}}^{\ast }=0.48\pm 0.23$. However, the prediction accuracy of the vector-oriented approaches drops less than 10% only when the number of factors varying in the range $20\le F\le 100$; the tensor-oriented methods remain within 90% of the optimal correlation in the range $20\le F\le 600$. The prediction accuracy results provided by the tensor-oriented approaches significantly outperform the vector-oriented ones for $F\ge 200$ (ANOVA test, significance level $\alpha =0.05$).

The cross-validation procedure^56^ is a state-of-the-art approach to estimate the number of factor hyper-parameters for the PLS-family methods, but it is time and memory intensive. Moreover, its direct application to online data flows is impossible. In addition, any predefined and fixed value of the hyper-parameter could be inappropriate because it should be regularly re-estimated to follow possible data variations over time. The Recursive-Validation procedure proposed here allows online estimates of the parameter values without significant additional computational time expenses.

Figure 6A demonstrates the prediction accuracy of the REW-NPLS model (identified with a predefined optimal number of factors ${F}^{\ast }=200$) compared to the REW-NPLS coupled with Recursive-Validation procedure for estimation of factor numbers. Both approaches showed equivalent prediction accuracies as follows from the experiments. Moreover, when the number of batches for model calibration is less than 25, the use of constant ${F}^{\ast }$ leads to overfitting in small training sets. However, the Recursive-Validation procedure adjusts ${F}^{\ast }$ value to optimize the prediction accuracy. Figure 6B demonstrates that the number of factors estimated by the Recursive-Validation procedure is significantly less than ${F}^{\ast }$ as estimated offline on the entire dataset. This additionally reduces the possibility of overfitting.

When all 70 batches are processed, ${F}_{{\rm{RV}}}^{\ast }=57\ll {F}^{\ast }=200$, the difference in prediction accuracies is insignificant (ANOVA test, significance level $\alpha =0.05$). Table 2 compares cross-validation and Recursive-Validation approaches on the MEG data set. The difference in the estimated optimal values of the factor numbers ${F}_{{\rm{RV}}}^{\ast }$ and ${F}_{{\rm{CV}}}^{\ast }$, as well as the corresponding values of the correlations on the test sets, are statistically insignificant (ANOVA test, significance level $\alpha =0.05$). Moreover, in comparison with the cross-validation method, the number of the factors provided by the Recursive-Validation approach is slightly smaller (${F}_{{\rm{RV}}}^{\ast }=5$ vs. ${F}_{{\rm{CV}}}^{\ast }=6$), whereas the correlation is essentially the same (${r}_{{\rm{RV}}}^{\ast }=0.29$ vs. ${r}_{{\rm{CV}}}^{\ast }=0.27$) for both left- and right index fingers movements. This could be explained by additional robustness of the Recursive-Validation procedure.

For tensor-based approaches, Influence Analysis can assess the weights of the temporal, frequency, and spatial modalities on the predictive model. Figure 7 represents the evaluation of the REW-NPLS model with Recursive-Validation of ${F}^{\ast }$ in time and its comparison with REW-NPLS model calibrated on the complete dataset with an optimal number of factors ${F}^{\ast }$ on the ECoG data.

For the REW-NPLS algorithm with Recursive-Validation, the models identified on batches 10, 25, 40, 55, and 70 (corresponding to 15%, 36%, 57%, 79%, and 100% of the training set) are considered. For the frequency modality, the weight distributions demonstrate the same behavior for all the models: the maximum peaks are at 10, 30, and 90 Hz. In the temporal modality, the weights of the elements tend to increase until 100 ms before the motion moment for both the online and offline models. In the spatial modality, for the REW-NPLS model, the set of the most relevant electrodes (10, 6, 8, and 15) is defined on the batch number 40 and does not change after; however, the weights of the electrodes become more equilibrate with time. For the offline model, the same electrodes 10, 6, 8, and 15 are the most informative.

Figure 8 represents applications of the Influence Analysis to the models identified by the REW-NPLS algorithm with the Recursive-Validation of ${F}^{\ast }$ in the MEG experiments. The results are prepared for the left and right finger self-paced movements and are averaged over 8 recordings carried out in 4 subjects. In the frequency modality, there is a maximum around 20 Hz for both fingers. The frequency bands above 50 Hz are less informative. This corresponds to the quality of the MEG-data recording system. In the temporal modality—similar to the case of the ECoG-based experiments—the weights of the elements tend to increase until the motion moment. In the spatial modality, the localization of the informative electrodes for the left and right finger correspond to the motor zone of the cortex^71^. For the left finger, the most informative electrodes are 1143, 1112, 1133, 1132, and 1142; whereas for the right finger, the most informative electrodes are 0442, 0433, 0712, 0443, and 0423 (according to the Elekta Neuromag system notation, see Fig. 4A).

The main limitation of the proposed REW-NPLS approach is its memory consumption, because it stores the covariance tensors in the memory. At the same time, the memory used by the algorithm is constant and does not change over time. The limitation of the Recursive-Validation algorithm is the need to compute the model for ${F}_{{\rm{\max }}}$ number of factors. This could be significantly greater than the optimal number ${F}_{{\rm{RV}}}^{\ast }$. However, ${F}_{{\rm{\max }}}$ can decrease with time if ${F}_{{\rm{RV}}}^{\ast }\ll {F}_{{\rm{\max }}}$ is constantly observed.

In parallel to a set of neuroscience tasks, such as BCIs, fMRI analysis, etc., the proposed REW-NPLS and the Recursive-Validation algorithms could be applied to a wide range of tasks where adaptive modeling of high dimension tensor flows is necessary. Examples include dynamic analysis of images and video sequences, adaptive monitoring of complex industrial processes, etc.

## Perspectives

The next step of the study is application of the proposed methods implemented in real-time operating software in the clinical BCI in tetraplegic subjects. Online adjustment provided by the REW-NPLS algorithm allows application of the closed-loop paradigm when the user immediately receives feedbacks from the system to his/her movement imaginations reflected in the brain activity. This will allow adaptation of the human behavior in parallel with adjustment of the BCI decoder. This should result in efficiency of the BCI system. Within the framework of the CEA-LETI-CLINATEC^®^ BCI project, the fully-implantable device WIMAGINE^®^^72^ for chronic measurement and wireless transmission of ECoG data is currently developed. The full body exoskeleton EMY^®^^73^ is designed to let the tetraplegic subjects possibility control the exoskeleton fragments in real-time. The clinical protocol named “Brain Computer Interface: Neuroprosthetic Control of a Motorized Exoskeleton” was authorized from the French regulatory agencies to start with five patients including bilateral implantation of 2 WIMAGINE^®^ implants per patient (https://clinicaltrials.gov/ct2/show/NCT02550522).

## Electronic supplementary material

Supplementary Information
