# Supplementary material for: Recursive Exponentially Weighted N-way Partial Least Squares Regression with Recursive-Validation of Hyper-Parameters in Brain-Computer Interface Applications
Source: Sci Rep. 2017 Nov 24;7:16281. doi: 10.1038/s41598-017-16579-9 (PMC5701264; doi:10.1038/s41598-017-16579-9)
Supplement: Supplementary file 1 — Supplementary Information [file 41598_2017_16579_MOESM1_ESM.pdf]

Recursive Exponentially Weighted N-way Partial Least Squares Regression with Recursive-Validation of Hyper-Parameters in Brain-Computer Interface Applications

Andrey Eliseyev[1*], Vincent Auboiroux[1], Thomas Costecalde[1], Lilia Langar[2], Guillaume Charvet[1], Corinne Mestais[1], Tetiana Aksenova[1], Alim-Louis Benabid[1]

[1] Univ. Grenoble Alpes, CEA, LETI, CLINATEC, MINATEC Campus, 38000 Grenoble, France

[2] Centre Hospitalier Universitaire Grenoble Alpes, 38700 La Tronche, France

[*] Corresponding author: eliseyev.andrey@gmail.com

Appendix A

REW-NPLS algorithm

Input: $\underline{\mathbf{X}}^{(t)}, \underline{\mathbf{Y}}^{(t)}, \underline{\mathbf{C}}_{\mathbf{XX}}^{(t-1)}, \ \underline{\mathbf{C}}_{\mathbf{XY}}^{(t-1)}, \{\mathbf{w}_f^1, \ldots, \mathbf{w}_f^n\}^{(t-1)}$

forgetting coefficient λ,

maximum number of factors $F_{\max}$.

Output: $\underline{\tilde{\mathbf{B}}}^{(t)}, \underline{\mathbf{Y}}_0^{(t)}, \underline{\mathbf{C}}_{\mathbf{XX}}^{(t)}, \underline{\mathbf{C}}_{\mathbf{XY}}^{(t)}, \{\mathbf{w}_f^1, \ldots, \mathbf{w}_f^n\}^{(t)}$.

1. $F_{\mathrm{RV}}^* = \mathbf{RecursiveValidation}\left(\underline{\mathbf{X}}^{(t)}, \underline{\mathbf{Y}}^{(t)}\right)$ — % F_{RV}^* provides the minimum error, taking into account current and previous datasets
2. $\left\{\underline{\mathbf{X}}_{\mathrm{Norm}}^{(t)}, \underline{\mathbf{Y}}_{\mathrm{Norm}}^{(t)}, \underline{\boldsymbol{\mu}\mathbf{X}}, \underline{\boldsymbol{\mu}\mathbf{Y}}, \underline{\boldsymbol{\sigma}\mathbf{X}}, \underline{\boldsymbol{\sigma}\mathbf{Y}}\right\} = \mathbf{Normalization}\left(\underline{\mathbf{X}}^{(t)}, \underline{\mathbf{Y}}^{(t)}\right)$ — % normalization of the new data
3. $\underline{\mathbf{C}}_{\mathbf{XX}}^{(t)} = \lambda \underline{\mathbf{C}}_{\mathbf{XX}}^{(t-1)} + \underline{\mathbf{X}}_{\mathrm{Norm}}^{(t)} \times_1 \underline{\mathbf{X}}_{\mathrm{Norm}}^{(t)}$ — % updating of the covariance tensor
4. $\underline{\mathbf{C}}_{\mathbf{XY}}^{(t)} = \lambda \underline{\mathbf{C}}_{\mathbf{XY}}^{(t-1)} + \underline{\mathbf{X}}_{\mathrm{Norm}}^{(t)} \times_1 \underline{\mathbf{Y}}_{\mathrm{Norm}}^{(t)}$
5. $\mathbf{C}_{\mathbf{XX}}^{(t)} = \mathbf{Reshape}\left(\underline{\mathbf{C}}_{\mathbf{XX}}^{(t)}\right) \in \mathbb{R}^{(I_1 \cdot \ldots \cdot I_n) \times (I_1 \cdot \ldots \cdot I_n)}$ — % reshape tensor to matrix
6. $\mathbf{C}_{\mathbf{XY}}^{(t)} = \mathbf{Reshape}\left(\underline{\mathbf{C}}_{\mathbf{XY}}^{(t)}\right) \in \mathbb{R}^{(I_1 \cdot \ldots \cdot I_n) \times (J_1 \cdot \ldots \cdot J_m)}$
7. $\mathbf{P} = \mathbf{0} \in \mathbb{R}^{(I_1 \cdot \ldots \cdot I_n) \times F_{\max}}$, $\mathbf{R} = \mathbf{0} \in \mathbb{R}^{(I_1 \cdot \ldots \cdot I_n) \times F_{\max}}$, $\mathbf{Q} = \mathbf{0} \in \mathbb{R}^{(J_1 \cdot \ldots \cdot J_m) \times F_{\max}}$ — % initialization of the matrix **P**, **R**, **Q** with **0**
8. for $f = 1, \ldots, F_{\max}$
9. if $m > 1$: $\tilde{\mathbf{q}} = \mathbf{eig}\left({\mathbf{C}_{\mathbf{XY}}^{(t)}}^T \mathbf{C}_{\mathbf{XY}}^{(t)}\right)$, else $\tilde{\mathbf{q}} = \mathbf{C}_{\mathbf{XY}}^{(t)}$ — % the eigenvector with the largest eigenvalue
10. $\underline{\mathbf{C}} = \mathbf{Reshape}\left(\mathbf{C}_{\mathbf{XY}}^{(t)} \tilde{\mathbf{q}}\right) \in \mathbb{R}^{I_1 \times \ldots \times I_n}$ — % reshape vector to tensor
11. $\{\mathbf{w}_f^1, \ldots, \mathbf{w}_f^n\}^{(t)} = \mathbf{PARAFAC}\left(\underline{\mathbf{C}}, \{\mathbf{w}_f^1, \ldots, \mathbf{w}_f^n\}^{(t-1)}\right)$ — % PARAFAC decomposition from the initial approximation
12. $\underline{\mathbf{w}} = \mathbf{w}_f^1 \circ \ldots \circ \mathbf{w}_f^n$ — % tensor formation
13. $\mathbf{w} = \mathbf{Reshape}\left(\underline{\mathbf{w}}\right) \in \mathbb{R}^{(I_1 \cdot \ldots \cdot I_n)}$ — % reshape tensor to vector

14. $\mathbf{w} = \mathbf{w}/\lVert\mathbf{w}\rVert$	% normalization
15. $\mathbf{r} = \mathbf{w}$	
16. for $k = 1, \ldots, f-1$	
17. $\quad \mathbf{r} = \mathbf{r} - (\mathbf{P}(:,k)^T\mathbf{w})\mathbf{R}(:,k)$	
18. end for	
19. $\tau = \mathbf{r}^T\mathbf{C}_{\mathbf{XX}}^{(t)}\mathbf{r}$	
20. $\mathbf{p} = \left(\mathbf{r}^T\mathbf{C}_{\mathbf{XX}}^{(t)}\right)^T/\tau$, $\mathbf{q} = \left(\mathbf{r}^T\mathbf{C}_{\mathbf{XY}}^{(t)}\right)^T/\tau$	
**21.** $\mathbf{C}_{\mathbf{XY}}^{(t)} = \mathbf{C}_{\mathbf{XY}}^{(t)} - \tau\mathbf{pq}^T$	
**22.** $\mathbf{Q}(:,f) = \mathbf{q}$, $\mathbf{P}(:,f) = \mathbf{p}$, $\mathbf{R}(:,f) = \mathbf{r}$	
**23.** $\mathbf{B}^f = \mathbf{R}(:,1{:}f)\mathbf{Q}(:,1{:}f)^T$	% matrix of the regression coefficients for the current f
24. $\underline{\mathbf{B}}^f = \mathbf{Reshape}(\mathbf{B}^f) \in \mathbb{R}^{I_1\times\ldots\times I_n\times J_1\times\ldots\times J_m}$	% reshape matrix to tensor
**25.** $\underline{\widetilde{\mathbf{B}}}^f = \underline{\mathbf{B}}^f\underline{\boldsymbol{\sigma}\mathbf{Y}}/\underline{\boldsymbol{\sigma}\mathbf{X}}$, $\underline{\mathbf{Y}}_0^f = \underline{\boldsymbol{\mu}\mathbf{Y}} - \underline{\boldsymbol{\mu}\mathbf{X}}\underline{\widetilde{\mathbf{B}}}^f$	% the regression coefficients and bias for the non-normalized data (see the **Normalization** algorithm)
end for	
26. $\underline{\widetilde{\mathbf{B}}}^{(t)} = \underline{\widetilde{\mathbf{B}}}^{F^*_{\mathrm{RV}}}$, $\underline{\mathbf{Y}}_0^{(t)} = \underline{\mathbf{Y}}_0^{F^*_{\mathrm{RV}}}$	% the optimal model to be used for prediction

- The n-mode vector product of tensor "$\times_n$"; see [57].
- PARAFAC algorithm description; see [32].
- The vector outer product "$\circ$"; see [57].

Appendix B

Normalization algorithm

Input:	$\underline{\mathbf{X}}^{(t)} \in \mathbb{R}^{N_t\times I_1\times\ldots\times I_n}$,
	$\underline{\mathbf{Y}}^{(t)} \in \mathbb{R}^{N_t\times J_1\times\ldots\times J_m}$,

	$N^{\mathrm{eff}(t-1)}$, $\underline{\mathbf{S}}_{\mathbf{X}}^{\mathrm{eff}(t-1)}$, $\underline{\mathbf{SS}}_{\mathbf{X}}^{\mathrm{eff}(t-1)}$, $\underline{\mathbf{S}}_{\mathbf{Y}}^{\mathrm{eff}(t-1)}$, $\underline{\mathbf{SS}}_{\mathbf{Y}}^{\mathrm{eff}(t-1)}$,
	forgetting coefficient λ.
Output:	$\underline{\mathbf{X}}_{\mathrm{Norm}}^{(t)}$, $\underline{\mathbf{Y}}_{\mathrm{Norm}}^{(t)}$
	$N^{\mathrm{eff}(t)}$, $\underline{\mathbf{S}}_{\mathbf{X}}^{\mathrm{eff}(t)}$, $\underline{\mathbf{SS}}_{\mathbf{X}}^{\mathrm{eff}(t)}$, $\underline{\mathbf{S}}_{\mathbf{Y}}^{\mathrm{eff}(t)}$, $\underline{\mathbf{S}}_{\mathbf{Y}}^{\mathrm{eff}(t)}$,
	$\underline{\boldsymbol{\mu}\mathbf{X}}$, $\underline{\boldsymbol{\sigma}\mathbf{X}}$, $\underline{\boldsymbol{\mu}\mathbf{Y}}$, $\underline{\boldsymbol{\sigma}\mathbf{Y}}$.

1. $N^{\mathrm{eff}(t)} = \lambda N^{\mathrm{eff}(t-1)} + N_t$ % effective number of points

2. $\underline{\mathbf{S}}_{\mathbf{X}}^{\mathrm{eff}(t)} = \lambda \underline{\mathbf{S}}_{\mathbf{X}}^{\mathrm{eff}(t-1)} + \underline{\mathbf{X}}_t \times_1 \mathbf{1}^{N_t},$

3. $\underline{\mathbf{S}}_{\mathbf{Y}}^{\mathrm{eff}(t)} = \lambda \underline{\mathbf{S}}_{\mathbf{Y}}^{\mathrm{eff}(t-1)} + \underline{\mathbf{Y}}_t \times_1 \mathbf{1}^{N_t}$

4. $\underline{\boldsymbol{\mu}\mathbf{X}} = \frac{\underline{\mathbf{S}}_{\mathbf{X}}^{\mathrm{eff}(t)}}{N^{\mathrm{eff}(t)}}, \underline{\boldsymbol{\mu}\mathbf{Y}} = \frac{\underline{\mathbf{S}}_{\mathbf{Y}}^{\mathrm{eff}(t)}}{N^{\mathrm{eff}(t)}}$ % mean$(\underline{\mathbf{X}})$, mean$(\underline{\mathbf{Y}})$

5. $\underline{\mathbf{SS}}_{\mathbf{X}}^{\mathrm{eff}(t)} = \lambda \underline{\mathbf{SS}}_{\mathbf{X}}^{\mathrm{eff}(t-1)} + \underline{\mathbf{X}}_t{}^{*2} \times_1 \mathbf{1}^{N_t}$

6. $\underline{\mathbf{SS}}_{\mathbf{Y}}^{\mathrm{eff}(t)} = \lambda \underline{\mathbf{SS}}_{\mathbf{Y}}^{\mathrm{eff}(t-1)} + \underline{\mathbf{Y}}_t{}^{*2} \times_1 \mathbf{1}^{N_t}$

7. $\underline{\boldsymbol{\sigma}\mathbf{X}} = \sqrt{\frac{\underline{\mathbf{SS}}_{\mathbf{X}}^{\mathrm{eff}(t)} - \left(\underline{\mathbf{S}}_{\mathbf{X}}^{\mathrm{eff}(t)}\right)^{*2} / N^{\mathrm{eff}(t)}}{N^{\mathrm{eff}(t)} - 1}}$ % std$(\underline{\mathbf{X}})$

8. $\underline{\boldsymbol{\sigma}\mathbf{Y}} = \sqrt{\frac{\underline{\mathbf{SS}}_{\mathbf{Y}}^{\mathrm{eff}(t)} - \left(\underline{\mathbf{S}}_{\mathbf{Y}}^{\mathrm{eff}(t)}\right)^{*2} / N^{\mathrm{eff}(t)}}{N^{\mathrm{eff}(t)} - 1}}$ % std$(\underline{\mathbf{Y}})$

9. $\underline{\mathbf{X}}_{\mathrm{Norm}}^{(t)} = \left(\underline{\mathbf{X}}^{(t)} - \underline{\boldsymbol{\mu}\mathbf{X}}\right) / \underline{\boldsymbol{\sigma}\mathbf{X}}$ % element-wise normalization of the tensor $\underline{\mathbf{X}}$

10. $\underline{\mathbf{Y}}_{\mathrm{Norm}}^{(t)} = \left(\underline{\mathbf{Y}}^{(t)} - \underline{\boldsymbol{\mu}\mathbf{Y}}\right) / \underline{\boldsymbol{\sigma}\mathbf{Y}}$ % element-wise normalization of the tensor $\underline{\mathbf{Y}}$

Appendix C

Recursive Validation algorithm

Input:	$\underline{\mathbf{X}}^{(t)}$, $\underline{\mathbf{Y}}^{(t)}$, $\{\underline{\widetilde{\mathbf{B}}}^f, \underline{\mathbf{Y}}_0^f\}_{f=1}^{F_{\max}}$, $\left\{e_f^{(t-1)}\right\}_{f=1}^{F_{\max}}$

forgetting coefficient γ.

Output: $F^*_{(t)}, \left\{e_f^{(t)}\right\}_{f=1}^{F_{\max}}$.

1. for $f = 1, \ldots, F_{\max}$

2. $\underline{\hat{\mathbf{Y}}}^f = \underline{\mathbf{X}}^{(t)} \underline{\tilde{\mathbf{B}}}^f + \underline{\mathbf{Y}}_0^f$ % estimation of the prediction on the new data with the old model

3. $e_f^{(t)} = \gamma e_f^{(t-1)} + \mathbf{ERROR}\left(\underline{\hat{\mathbf{Y}}}^f, \underline{\mathbf{Y}}^{(t)}\right)$ % error between predicted and observed values on the new data, plus previous errors

4. end for

5. $F^*_{(t)} = \underset{f}{\operatorname{argmin}}\left\{e_f^{(t)}\right\}_{f=1}^{F_{\max}}$ % find number of factors, providing the minimal error
